# The impact of p16^ink4a^ positivity in invasive vulvar cancer on disease-free and disease-specific survival, a retrospective study

**DOI:** 10.1007/s00404-020-05431-7

**Published:** 2020-01-22

**Authors:** Lisa Gensthaler, Elmar A. Joura, Laia Alemany, Reinhard Horvat, Silvia de Sanjosé, Sophie Pils

**Affiliations:** 1 Department of Gynecology and Obstetrics, Medical University of Vienna, Waehringer Guertel 18-20, 1090 Vienna, Austria; 2 Unit of Infections and Cancer, Cancer Epidemiology Research Program, Catalan Institute of Oncology, IDIBELL, L’Hospitalet de Llobregat, Barcelona, Spain; 3 Department of Pathology, Medical University of Vienna, Vienna, Austria

**Keywords:** Vulvar cancer, HPV, p16^ink4a^, Survival, Prognosis

## Abstract

**Purpose:**

To evaluate HPV and p16^ink4a^ status as prognostic factors in patients with invasive vulvar cancer.

**Methods:**

Retrospective analysis of disease-free (DFS) and disease-specific survival (DSS) of patients with invasive vulvar cancer at a single tertiary care center. Histology, HPV and p16^ink4a^ status were evaluated in the context of a global multicenter trial. Logistic regression models were performed to identify the impact of p16^ink4a^ positivity.

**Results:**

135 patients were included in the analysis. 32 (23.7%) showed a p16^ink4a^ expression of over 25%. Disease-free and disease-specific survival was longer in p16^ink4a^ positive patients (23 vs. 10 months, *p* = 0.004, respectively, 29 vs. 21 months, *p* = 0.016). In multivariate analysis, p16^ink4a^ positivity was an independent parameter for DFS (*p* = 0.025, HR: 2.120 (1.100–4.085)), but not for DSS (*p* = 0.926, HR: 1.029 (0.558–1.901), in contrast to age and tumor stage.

**Conclusions:**

Age and tumor stage negatively affect survival. However, disease-free survival is significantly longer in patients with p16^ink4a^ positive invasive vulvar cancer.

## Introduction

Over the past two decades, the incidence rates of invasive vulvar cancer, which is responsible for about 5% of all gynecological cancers, increased continuously. Especially in women younger than 50 years of age, the incidence rates almost tripled over the past decades [1–3]. Especially the influence of HPV-positivity and p16^ink4a^ expression on patient’s outcome is the main focus nowadays [4]. In the largest multicenter study, including 1709 cases in 39 countries from five continents, de Sanjosé et al. were able to identify Human papilloma virus (HPV) infection in approximately 25% of invasive vulvar cancer (IVC) cases [5, 6]. Similar findings could be shown in a Scottish single-center study, where Wakeham et al. reported a prognostic benefit on the clinical outcome of HPV-positive vulvar cancer [7]. In a meta-analysis including 7.721 patients, Zhang et al. supported these results [8]. In a recently published Dutch study, Hinten et al. described the combination of HPV and p16^ink4a^ positivity as a favorable prognostic factor in IVC [9]. P16^ink4a^ overexpression, defined as at least 25% of cells with nuclear or cytoplasmic staining, is an indicator of HPV associated tumors. Transient HPV infections can be excluded and only tumors with HPV as the primary cause of the oncologic process remain [10–12]. We present the clinical Austrian data nested in the global multicenter study and combined this with clinical outcome data. While the presence of lymph node metastasis is the most important predictive parameter for survival, the p16^ink4a^ status may strongly influence the patient’s outcome, which is already established in oropharyngeal cancer [7]. Age at diagnosis, initial treatment option, histological groups, clinical outcome, recurrence and regression rates and patient’s comorbidities were evaluated retrospectively. The aim of the study was to evaluate the p16^ink4a^ positivity and HPV status on the clinical outcome in IVC.

## Material and methods

Austria was one of 39 countries participating in the international collaborative study [4]. Patients diagnosed with primary IVC at the Department for Gynecological Oncology of the Medical University of Vienna between 1995 and 2012 were enrolled in this cross-sectional period-prevalence study on archival specimens. The paraffin-embedded blocks of archival histological specimens were sent to the study center at the Institut Catalan di Oncologia (ICO) in Barcelona for further evaluation. Initially, 204 samples of vulvar neoplasia and control samples were sent to Barcelona for histopathological evaluation. 177 samples were classified as suitable for HPV-testing. According to protocol, specimen were reviewed and classified, HPV-type and expression of the tumor suppressor protein p16^ink4a^ was evaluated. A sensitive assay, using SPF10 broad spectrum primers PCR and DEIA (DNA enzyme immunoassay) was used for HPV–DNA detection. Positive samples were subsequently analyzed by SPF10 PCR/DEIA/LIPA25 (RHA Kit HPV SPF10-LiPA25, version 1 by Labo Biomedical Products, Rijswijk, The Netherlands), which is a reverse hybridization technique that detects 25 high-risk (HR) and low-risk (LR) HPV types (6, 11, 16, 18, 31, 33, 34, 35, 39, 40, 42, 43, 44, 45, 51, 52, 53, 54, 56, 58, 59, 66, 68, 70, 74). Furthermore, IVC cases were tested for cyclin-dependent kinase-4 inhibitor (p16^ink4a^), which is reported to be overexpressed in at least 90% of HPV-related VIN and IVC cases. Therefore, CINtec^®^ PLUS Cytology Kit by Roche (clone E6H4, ROCHE MTM Laboratories, Heidelberg, Germany) was used to detect p16^ink4a^ in the invasive vulvar cancer cases. A case was considered to be positive if more than 25% of invasive cancer cells showed a diffuse overexpression [4, 5, 13]. The clinical data were collected and evaluated retrospectively at our institution. Follow-up and clinical outcome were available for 135 eligible patients. Data was collected from June 1993 until January 2016. Before the study was initiated, it was approved by the Ethics Committee of the Medical University of Vienna (IRB approval number: 1997/2015, approved on May 20th, 2016). Since this study was a retrospective analysis, no informed consent from patients was required by the ethics committee. Patient’s records were anonymized prior to analysis.

### IVC management

All patients included were managed by gynecologic oncologists. Predefined uniform criteria for surgical procedure terminology, pathologic variables, and sites of recurrence were used. Based on the FIGO 2009 classification system, disease staging was performed [14]. Depending on the tumor’s extent and the physician’s assessment, patients underwent surgery. In case of positive lymph nodes, adjuvant radiotherapy was performed.

For the first 2 years, clinical examination was performed every 3 months, followed by 6 months intervals until the completed fifth year, then by annually consultations. At every follow up check, the patient received a vulvoscopy, vagino-rectal palpation and groin inspection. Furthermore, serum squamous cell carcinoma antigen (SCC) was evaluated. Biopsy and or computed tomography were performed when recurrent disease was suspected. Based on post-mortem results, patient’s death and causes of death were documented.

### Statistical analysis

Patient’s data was divided in two different groups (group1/group2), assuming that cases in which HPV–DNA is detected without overexpression of p16^ink4a^ could represent a transient infection with no role in carcinogenesis. Group 1 represented patients with HPV-positive and HPV-negative IVC with a p16^ink4a^ expression in less than 25% (< 25% = negative) and group 2 consisted of HPV-positive and HPV-negative IVC with a concomitant overexpression of p16^ink4a^ (≥ 25%). Values are shown as mean values with standard deviation (SD). To compare HPV-positivity with clinic-pathological parameters, *t* tests were performed. *P* values of < 0.05 were considered statistically significant. With respect to overall and disease-free survival, differences between groups were tested using the log-rank test and are presented as Kaplan–Meier survival curves. Multivariable analysis was performed using a Cox regression model including patient’s age (mean, IQR), p16^ink4a^ expression and tumor stage (FIGO III and IV vs. FIGO I and FIGO II) as independent variables. Furthermore, FIGO I and II adapted analysis was performed via *t* test and is presented as Kaplan–Meier survival curves as well. Statistical analyses were performed using SPSS 25.0 for MAC (SPSS 25.0, IBM Inc., Armonk, NY). *Collected data:* age at diagnosis, histopathological grading, initial therapy, HPV-subtype analysis, p16^ink4a^ expression, FIGO classification, disease-free survival, disease-specific survival, nicotine abuse, coexistent lichen sclerosus or VIN, previous malignant disease, comorbidities.

## Results

135 patients with invasive vulvar cancer were analyzed. Patients were grouped by their p16^ink4a^ status to exclude transient HPV infections (Table 1). Patient characteristics are shown in Table 2. The median age at diagnosis of patients in group 1 was 71, ranging from 58–84 years (IQR), whereas patients from group 2 were by median 64 years of age (IQR: 47–81 years). Patients of group 2 were significantly younger (*p* < 0.001). In 30 (22.2%) of the evaluated histological specimen, HPV was detected. HPV 16 was the primarily diagnosed HPV-subtype and detected in 27 cases (77%). HPV 31 was detected in two cases (6.7%) and HPV 33 (3.3%) in one. In 20 (66.7%) of those HPV-positive cases, an overexpression (≥ 25%) of the tumor-suppressor-protein p16^ink4a^ was detected. Contrary, only 12 (11.4%) of HPV-negative IVC presented an overexpression of p16^ink4a^ (Table 1).

**Table 1:** Group classification depending on HPV and p16^ink4a^ expression in patients with invasive vulvar cancer

	Group 1 *n* = 103 (76.3%)	Group 2 *n* = 32 (23.7%)
HPV-positive, p16^ink4a^ ≥ 25%	0 (0)	20 (62.5)
HPV-negative, p16^ink4a^ ≥ 25%	0 (0)	12 (37.5)
HPV-positive, p16^ink4a^ < 25%	10 (9.7)	0 (0)
HPV-negative, p16^ink4a^ < 25%	93 (90.3)	0 (0)

Group 1: p16^ink4a^ < 25%; Group 2: p16^ink4a^ ≥ 25%

**Table 2:** Patient’s characteristics

	Group 1 *n* = 103 (76.3%)	Group 2 *n* = 32 (23.7%)	*p* value
Age in years, median (IQR)	71 (58–84)	64 (47–81)	0.001^1^
Initial treatment			0.547^2^
Surgery (%)	97 (94.2)	31 (96.9)	
Radiation (%)	6 (5.8)	1 (3.1)	
FIGO			
I&II (%)	80 (77.7)	23 (71.9)	
III&IV (%)	23 (22.3)	9 (28.1)	
FIGO I&II vs. FIGO III&IV			0.501^2^
Grading			0.492^2^
1 (%)	37 (36)	8 (25)	
2 (%)	56 (54)	21 (66)	
3 (%)	10 (10)	3 (9)	
Smoking	*n* = 72	*n* = 19	0.421^2^
Yes (%)	13 (18)	5 (26)	
No (%)	59 (82)	14 (74)	
Disease-free survival in months, median (IQR)	10 (5–36)	23 (4–65.5)	0.004^3^
Disease-specific survival in months, median (IQR)	21 (8–87)	29.5 (4–116.5)	0.016^3^

Group 1: p16ink4a < 25%; Group 2: p16ink4a ≥ 25%

^a^*t* test, ^b^Chi-square test, ^c^log-rank

A coexistent lichen sclerosus was detected in 6 (4.4%) cases, all of them were HPV-negative. FIGO stages were well balanced between both groups (*p* = 0.501). Further subgroup analysis, only including patients at FIGO stage I and II was performed.

**Fig. 1:**
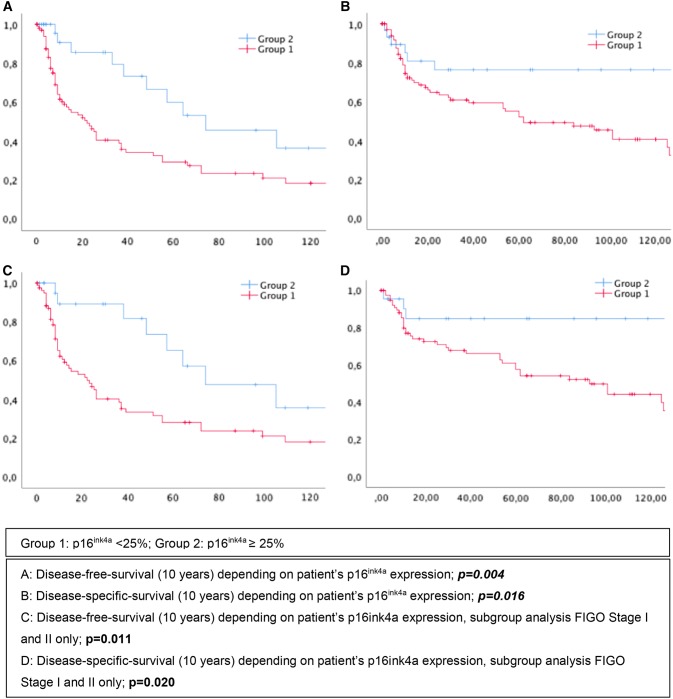
Kaplan Meier curves on disease-free and overall survival

76 (56.3%) patients suffered at least one relapse. Mean disease-free survival (DFS) in group 1 was 10 months (IQR: 5–36), whereas patients from group 2 had a relapse after 23 months (IQR: 4–65.5), *p* = 0.004 (Fig. 1a). Similar results could be shown regarding the patient’s disease-specific survival. Patients of group 2 lived in average 29 months after primary diagnosis (IQR: 4–116.5), whereas patients of group 1 only survived 21 months (IQR: 8–87), *p* = 0.016 (Fig. 1b). In multivariate analysis, p16^ink4a^ positivity still could be verified as an independent parameter regarding disease-free survival (*p* = 0.025, HR: 2.120 (1.100–4.085)) but not disease-specific survival (DSS) (*p* = 0.926, HR: 1.029 (0.558–1.901)). Results on multivariate analysis are presented in Table 3.

**Table 3:** Cox regression analysis of predictive markers in patients with invasive vulvar cancer

Parameter	Multivariate analysis Disease-free-survival		Multivariate analysis Disease-specific-survival	
	*p* value	HR (95% CI)	*p *value	HR (95%CI)
p16^ink4a^ ≥ 25%	0.025	2.120 (1.100;4.085)	0.926	1.029 (0.558;1.901)
Age	0.010	1.024 (1.006;1.043)	< 0.001	1.060 (1.038;1.082)
FIGO	0.984	1.007 (0.511;1.982)	0.001	2.413 (1.404;4.147)

FIGO I and II vs. FIGO III and IV

To have a closer look at patients with a favorable prognosis, FIGO stage I and II were analyzed separately (Table 4). Patients of group 1 (*n* = 80, 77.7%), still were significantly older (mean: 70.5 years (IQR: 57.4–83.6)), than patients of group 2 (mean: 63.1 years (IQR:46.0–79.5)), *p* = 0.028. Disease-free survival of group 2 still was significantly longer than in group 1; *p* = 0.011 (Fig. 1c). We obtained similar results regarding DSS; *p* = 0.020 (Fig. 1d). Group 2 could still be verified as an independent prognostic factor in multivariate analysis regarding DFS (*p* = 0.036, HR: 2.151 (1.053–4.393)), but not concerning DSS (*p* = 0.774, HR: 1.111 (0.541–2.285), Table 5).

**Table 4:** Patient’s characteristics, subgroup analysis FIGO stage I and II only

	Group 1 *n* = 80 (77.7%)	Group 2 *n* = 23 (22.3%)	*p* value
Age in years, median (IQR)	70.5 (57.4–83.6)	63.1 (46–79.5)	0.0281
Initial treatment			0.8982
Surgery (%)	76 (95)	22 (96)	
Radiation (%)	4 (5)	1 (4)	
Grading			0.321^2^
1 (%)	34 (36)	6 (25)	
2 (%)	40 (54)	14 (66)	
3 (%)	6 (10)	3 (9)	
Smoking	*n* = 72	*n* = 19	0.421^2^
Yes (%)	13 (18)	5 (26)	
No (%)	59 (82)	14 (74)	
Disease-free survival in months, median (IQR)	11.5 (6–38.5)	38(8–74)	0.011^3^
Disease-specific survival in months, median (IQR)	37.5(10–94.7)	46(10–119)	0.020^3^

Group 1: p16^ink4a^ < 25%; Group 2: p16^ink4a^ ≥ 25%

^a^*t* test, ^b^Chi-square test, ^c^log-rank

**Table 5:** Cox regression analysis of predictive markers in patients with invasive vulvar cancer with FIGO stage I and II

Parameter	Multivariate analysis Disease-free survival		Multivariate analysis Disease-specific survival	
	*p* value	HR (95% CI)	*p* value	HR (95%CI)
p16^ink4a^ ≥ 25%	0.036	2.151 (1.053;4.393)	0.774	1.111 (0.541;2.285)
Age	0.016	1.025 (1.005;1.045)	< 0.001	1.070 (1.043;1.098)

Furthermore, for sensitivity analysis, patients aged 80 years, or more were excluded in the subgroup analysis of FIGO stages I and II. Excluding the old ages as a possible bias, the difference between both groups for DFS and DSS remained statistically significant (*p* = 0.010 and *p* = 0.048, respectively).

## Discussion

In our dataset of 135 IVC, survival indicators, DFS and DSS, were significantly longer in p16^ink4a^ positive patients. In multivariate analysis, p16^ink4a^ positivity remained an independent favorable prognostic factor for DFS, whereas DSS was not affected. This study was a clinical sub-analysis of an international collaborative study, initiated by de Sanjosé, where 39 countries including our institution participated in that cross-sectional period-prevalence study on archival specimens, where more than 2000 IVC were histopathological analyzed [4]. The main advantage was the single-center analysis, using highly standardized and specified protocols. During a prolonged observational period of more than 10 years, patients were observed by a single group of specialized, oncologic gynecologists, providing the high quality of patient care and precise detection rate of IVC relapses. The study setting provided a reliable assessment of histology, HPV and p16^ink4a^ analysis linked to single-center clinical data with a long observational period, although retrospective design is an undeniable limitation. Due to the retrospective design, only data on initial smoking behavior are available, no continuous data were collected. In addition, data on vaccination status and quality of life are not available. Our results support the recent published study by Arians et.al., where p16^ink4a^ overexpression, which is as a marker for persistent HPV infections, seems to have a beneficial influence on disease-free and disease-specific survival of patients with IVC [15–17]. To be comparable with the recently published data, we replicated the group assignments and analysis according to Hinten’s recently published results. The difference in age of our patient cohorts was smaller than in the Dutch publication (71 vs. 64 years, compared to Hinten’s 72 vs. 55 years). Therefore, we can assume a similar state of health in both observed groups [9]. To evaluate the role of HPV and related markers, a sub-analysis of small tumors (FIGO I and II), was performed to reduce potential bias due to poor prognosis related to advanced stage that could be independent of HPV status. In the Dutch paper there was an uneven distribution of tumor stage with more FIGO stage III and IV disease in the p16^ink4a^ negative cohort which is a clinically relevant bias. 45% patient’s with p16^ink4a^ negative IVC were diagnosed in higher FIGO stage III and IV (*p* = 0.001) [9]. In our analysis, the FIGO stages at diagnosis (FIGO I and II vs. FIGO III and IV) were very well balanced (Table 2, *p* = 0.501). Although patients with p16^ink4a^ positive IVC were on average seven years younger than p16^ink4a^ negative controls and the first recurrence occurred after an average of 2 years instead of one year, in multivariate analysis DSS depends on younger age and lower FIGO stage but not on p16^ink4a^ status. Therefore, further research has to be done on data of relapse in IVC. Since there is no appropriate screening for VIN and IVC, elimination of HPV-related tumors is possible in countries with a good coverage of HPV vaccination [7, 13, 18–22]. In conclusion, higher age and tumor stage negatively affect survival. However, disease-free survival is significantly longer in patients with p16^ink4a^ positive invasive vulvar cancer. There is a caveat which needs further investigation: In case of a relapse, the mortality of the initially prognostic favorable p16^ink4a^ positive invasive vulvar cancer appears to be worse.
